# Modified Meyerhof approach for forecasting reliable ultimate capacity of the large diameter bored piles

**DOI:** 10.1038/s41598-022-12238-w

**Published:** 2022-05-20

**Authors:** M. E. Al-Atroush, A. M. Hefny, T. M. Sorour

**Affiliations:** 1 Engineering Management Department, College of Engineering, Prince Sultan University, Riyadh, Kingdom of Saudi Arabia; 2 Department of Civil and Environmental Engineering, United Arab Emirates University, Al-Ain, UAE; 3 Department of Civil Engineering, Faculty of Engineering, Ain Shams University, Cairo, Egypt

**Keywords:** Engineering, Civil engineering

## Abstract

The static loading test is undoubtedly the most reliable method for forecasting the ultimate capacity of the large diameter bored piles (LDBP). However, in-situ loading of this class of piles until reaching failure is practically seldom due to the large amount of settlement required for shaft and base mobilization. Therefore, many international design standards recommend either capacity-based or settlement-based methods to estimate the LDBP ultimate capacity in case of the impossibility of performing loading tests during the design phase. However, those methods are invariably associated with various degrees of uncertainty resulting from several factors, as evidenced in several comparative analyses available in the literature. For instance, the settlement-based method of the Egyptian code of practice (ECP 202/4) usually underestimates the ultimate capacity of LDBP. In contrast, Meyerhof's capacity-based method often overestimates the LDBP’s ultimate capacity. In this paper, a modified approach has been proposed to forecast the ultimate capacity of the LDBP. This approach was modified from Meyerhof’s classical formula (1976) through three fundamental stages. First, an assessment study was performed to evaluate the reliability of the estimated LDBP ultimate capacity using Meyerhof’s classical method. For this purpose, results of full scale loaded to failure loading LDBP test and related twenty-eight parametric numerical models with various pile geometrical and soil geotechnical parameters have been used. Based on the assessment study findings, the essential modifications were suggested in the second stage to adapt Meyerhof’s classic method. In the third stage, the results of several numerical models and in-situ loading tests were employed to assess the accuracy of the developed modified method. This study showed that Meyerhof's classical method overestimated the ultimate capacity of LDBP with an error percentage ranging from 14 to 46%. On the other side, the proposed modified approach has succeeded in estimating the ultimate capacity of loaded to failure in-situ LDBP test and twenty numerical LDBP models with error percentages ranging from 0.267 to 7.75%.

## Introduction

In line with the rising population density, structures’ height limits are being revisited and revised worldwide to maximize land use. Moreover, with the recent advancements in engineering technologies, the construction of higher and heavier structures has become more applicable. With that in mind, large-diameter bored piles (LDBP) were the most frequently employed foundation system for such cases due to their effectiveness in supporting heavy loads and minimizing settlement^1^. Nevertheless, most of the available methods for forecasting the ultimate capacity of the LDBP are invariably associated with various degrees of uncertainty, as reported in several assessment studies^2–7^.

In general, three types of methods are commonly used for estimating the ultimate capacity of LDBP at the design phase. First are the settlement-based methods that estimate the ultimate bearing resistance of the LDBP at a particular settlement value. Those methods generally define the nominal ultimate pile capacity (Q_ult_) using different percentages of the pile head settlement to diameter ratio (S_max_/d), such as 10%, according to Weltman^8^, 4% according to Kulhawy and Hirany^9^, and 5%, according to O'Neill and Reese^1^. Second are the correlation methods that estimate the pile's ultimate friction resistance using empirical correlations with the standard penetration (SPT) and the cone penetration (CPT) results (i.e., Eslami and Fellenius^10^ and Niazi and Mayne^11^). Lastly, analytical capacity-based methods mainly rely on geotechnical calculation models or analytical procedures to estimate the ultimate capacity of the LDBP (i.e., Meyerhof 1976).

Comprehensive discussions on the aforementioned settlement-based methods were carried out by Meyerhof^12^ and Charles et al.^13^; also, an assessment study was performed by Abu-Farsakh and Titi^14^ and Tawfik^15^ utilizing loading tests to evaluate the reliability of those methods. It was concluded that those methods might result in a conservative estimation of the ultimate pile capacity. On the other side, the negligence of the potentially existing variation of the in-situ soil properties along the pile shaft due to the boring process and the considered assumptions in geotechnical calculation models were the most reported primary sources of the associated uncertainty with the correlation empirical methods and the capacity-based methods, respectively^16–20^.

No doubt that the full-scale static pile loading test represents the most reliable methodology for estimating the ultimate capacity of the large diameter bored piles (LDBP)^21^. Several international geotechnical codes and foundation design standards (e.g.^22–24^; and many others) recommend this method to study and investigate the load transfer and failure mechanisms of this class of piles. However, in most cases, the obtained load-settlement curves from such tests conducted on LDBP tend to increase without reaching the failure point or an asymptote. Loading LDBP till reaching apparent failure is practically seldom because of the significant amount of pile settlement that is usually required for the full mobilization of the pile shaft and reaching the ultimate base resistances^1,25,26^. Huge test loads, and hence, high-capacity reaction systems should be used to accomplish the required enormous settlements. Thus, the targeted failure load may not always be practical to achieve, as reported in many case studies^27–29^.

Eid et al.^28^ performed a full-scale well-instrumented loading test for an LDBP implemented in multi-layered soil, with 1 m diameter and 34 m length. Although the soil profile included more than 15 m of soft soil and the LDBP was tested under an applied load equal to three times the design load (Based on ECP202/4^22^ design criteria), but the results of this in-situ loading test highlighted that the tested pile safely sustained a load of 300% of the design load without achieving the failure. Besides, the measured settlement under a load of 200% of the working load was less than the allowable settlement (Calculated using the ECP criteria). This may raise a question about the reliability of the suggested estimation methods by different international codes to forecast the allowable capacity of LDBP at the design phase or in case of impossibility to perform pile loading test.

With that in mind, the load transfer method is generally a simple analytical procedure that can be applied in many complex situations, such as variation in the sections along a pile shaft and an inhomogeneous layered soil system. In the same line, Meyerhof’s capacity-based classic formula is endorsed in several international design standards for estimating the ultimate pile capacity. Meyerhof^30^ developed his theory of bearing capacity of foundations based on the plastic theory by extending the previous analysis for surface footings to shallow and deep foundations in a uniform, cohesive material that exposed internal friction (c–Ø soil). Meyerhof^31^ utilized the empirical data obtained from field observations alongside some theoretical considerations in developing his classic formula for the bearing capacity of a pile in a soil possessing both cohesion and friction. As presented in Eq. (1), the foundation's physical characteristics and the soil's mechanical properties were represented in this formula through bearing capacity factors (N_c_, N_q,_ and N_ɣ_).

1 $$ {\text{P}}_{{{\text{ult}}}} = {\text{A}}_{{\text{s}}} \left( {{\text{c}}_{{\text{a}}} + {\text{K}}_{{\text{s}}}\upgamma \frac{{\text{L}}}{2}\tan\updelta } \right) + {\text{A}}_{{\text{b}}} \left( {{\text{cN}}_{{\text{c}}} +\upgamma {\text{LN}}_{{\text{q}}} +\upgamma \frac{{\text{D}}}{2}{\text{N}}_{\upgamma } } \right) $$

where A_s_: Shaft surface area; c_a_: soil adhesion per unit area; δ: friction angle of the soil on the shaft; K_s_: the earth pressure coefficient; L/2: critical depth; A_b:_ Base cross-sectional area; D: Pile diameter; c: soil cohesion; γ: Soil unit weight; N_c_, N_q,_ and N_γ_: bearing capacity factors depend on ϕ and the embedment depth ratio L/B.

Several assessment studies admitted the accuracy of the Meyerhof formula in forecasting a reliable pile ultimate capacity for the small diameter piles less than 60 cm. However, Al-Atroush et al.^32^ compared the field measurements of loaded to failure LDBP with a diameter of 1.30 m with the estimated ultimate pile capacity using Meyerhof’s capacity-based method and different settlement-based methods from several codes. It was concluded that for both drained and undrained conditions, Meyerhof’s method overestimated the ultimate capacity of the LDBP (with diameters greater than 60 cm). In contrast, different codes’ settlement-based criterions underestimated the LDBP ultimate capacity of the same case. Those results were also consistent with the findings of several other assessment studies^28,29,33–35^ for different pile loading tests with various large diameters and different sub-surfaces. Therefore, there is still a need for a theoretically sound method able to predict a reliable value for the ultimate capacity of large diameter bored piles (LDBP).

This paper aspires to propose an analytical approach able to estimate a reliable ultimate capacity for LDBP. Results of a comprehensive numerical parametric study are utilized to assess the reliability of Meyerhof's^31^ capacity-based method and several settlement-based methods in estimating the ultimate capacity of the LDBP with respect to the variation of the pile geometrical and soil geotechnical parameters. Several adaptations are proposed accordingly to enhance the reliability of Meyerhof’s method. In addition, in-situ LDBP tests and twenty numerical LDBP models are utilized to examine the accuracy of the modified Meyerhof method.

## Background and reference studies

Three studies’ findings were fundamentally incorporated to construct the hypothesis of this study. First is the field study of the Alzey bridge full-scale LDBP test. This LDBP was axially loaded to failure by Sommer and Hambach^36^. Second, the established numerical studies to simulate the behavior of the Alzey case history’s LDBP^32,37^. Third, the comprehensive parametric study^6^ that carried out to investigate the effect of different geometrical and geotechnical parameters on the ultimate capacity and settlement of the LDBP. The following sections give a brief about each of those three studies.

### Full scale loaded to failure LDBP in-situ test

**Figure 1:**
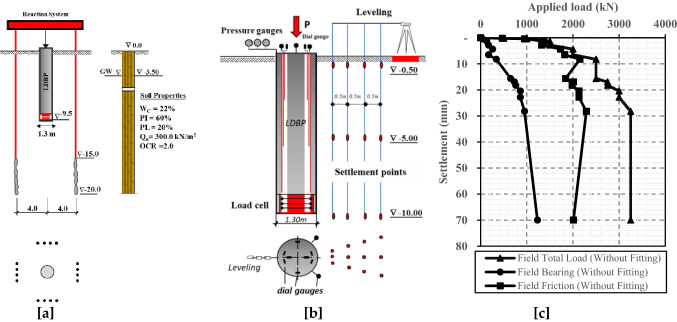
Large diameter bored pile loading to failure test (Modified from Sommer and Hammbach^36^). (**a**) Test arrangement and typical soil profile with mechanical properties. (**b**) Measuring devices and instrumentations. (**c**) Field measurements of the LDBP loading test.

Sommer and Hammbach^36^ carried out a full-scale and well-instrumented loading test at the location of Alzey bridge (Germany) to investigate the behavior of a large diameter bored pile (LDBP). The length and diameter of the investigated pile were 9.50 m and 1.30 m, respectively. Test setup and the soil characteristics at the site location are given in Fig. 1a. The instrumentations utilized in the LDBP loading test are described in Fig. 1b. This LDBP was installed in over consolidated stiff clay soil and subjected to axial loading cycles until achieving failure. Finally, the main measurements of the well-instrumented load test are summarized in Fig. 1c.

As shown in Fig. 1c, the significant increase in the measured settlement indicated apparent failure at the end of this test when the last load increment was applied on the pile head. More details of the loading test are available in Sommer and Hammbach^36^.

### Numerical calibration study

**Figure 2:**
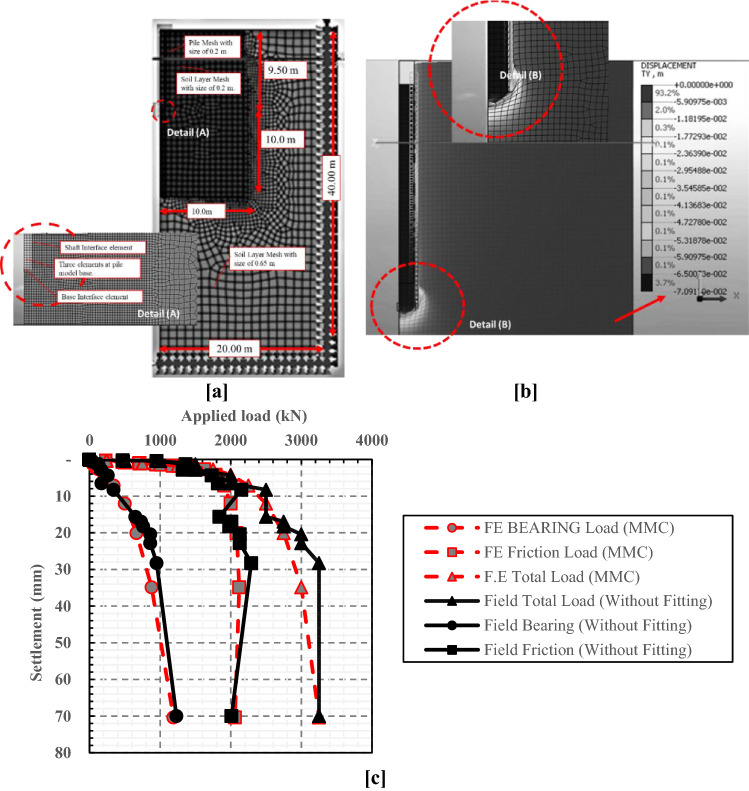
Numerical model established to simulate the response of the LDBP of the Alzey bridge case history (After Al-Atroush et al.^32^). (**a**) Details of boundary conditions. (**b**) Deformed shape of the finite element mesh under the Failure load. (**c**) Comparison between field measurements and the numerical results.

The numerical studies^32,37^ have been carried out to simulate the response of the LDBP of the Alzey bridge case history. Figure 2a shows the numerical model established to simulate the drained condition of over consolidated (OC) stiff clay soil. The micro-fissures associated with the OC stiff clay were the main reason behind using the drained condition to simulate the behavior of the soil in this case study. These micro-fissures usually provide avenues for local drainage; soil along fissures has softened (increased water content) and is softer than intact material. A more comprehensive discussion for using the drained condition with OC stiff clay is provided in Al-Atroush et al.^32^.

Three constitutive soil models have been utilized to simulate the drained condition of the overconsolidated (OC) stiff clay soil. It was found that for this case history, Modified Mohr–Coulomb (MMC) constitutive model was superior to the Mohr–Coulomb (MC) and the soft soil model (SS) in the simulation of the soil behavior^37,38^. Furthermore, the secant stiffness non-linear convergence method has been utilized to provide numerical stability required for software solvers to obtain convergence at substantial strain results (At failure). The performed sensitivity analyses highlighted the significant effects of the mesh size and geometry dimensions on the analysis results, and the optimum mesh size was accordingly obtained. Results of this calibration study showed an excellent agreement between finite element results and the in-situ measurements of both the pile load settlement and load transfer relationships, as presented in Fig. 2b and c.

**Figure 3:**
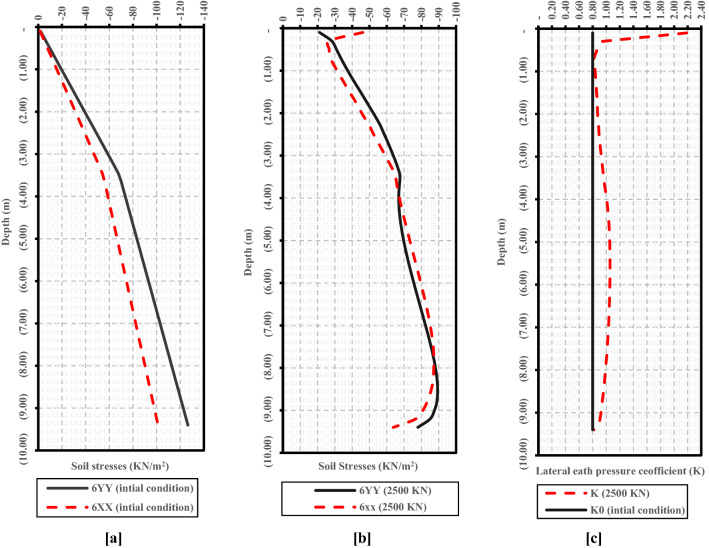
Finite element results of soil stress distribution with depth (After Ezzat et al.^37^). (**a**) Vertical and horizontal soil stress distribution with depth at the initial condition. (**b**) Vertical and horizontal soil stress distribution with depth after full mobilization. (**c**) Lateral earth pressure coefficient at the initial condition (K_0_) and after full mobilization.

The failure of the LDBP was explained by the arching action that occurred after the full shaft mobilization^37^. The study also revealed that arching action at the pile base causes a change in both soil’s vertical and horizontal stresses around the pile. As shown in Fig. 3, horizontal soil stresses values were less than vertical ones at the upper zone of the pile shaft length (along the first 4.0 m below the ground surface). However, at a distance of about 3.0 m (H/3) above the base, the horizontal soil stresses tended to be greater than the vertical ones. On the other hand, both vertical and horizontal stresses decreased at the pile base level, leading to an observed decrease in the unit skin friction results after full mobilization (Fig. 3b). It was also noted that at the full mobilization of side resistance, K values along the pile (Fig. 3c) were greater than the at-rest initial value (0.8) (Fig. 3a) except at the base of the pile, where a value equal to the at-rest value was observed. Moreover, the most significant values of K were observed near the ground surface.

Ezzat et al.^37^ also investigated the propagation of the plastic zone around the pile at the failure state. It was found that at the failure load, the plastic zone fully covered the whole length of the pile shaft interface. The formed plastic bulb around the pile base has a size of about four times of pile diameter (4D) and is extended below the pile base for a length of about three times (3D) of pile diameter.

### Parametric numerical study

**Figure 4:**
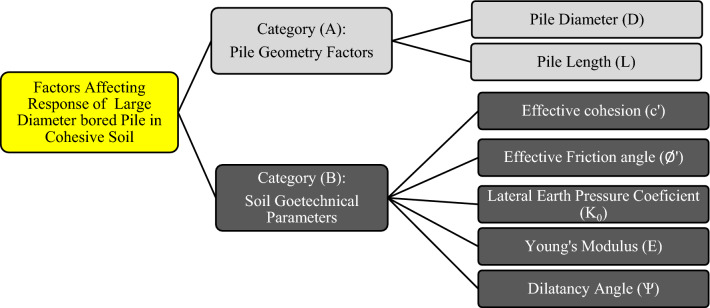
Factors affecting large diameter bored pile behavior (After Al-Atroush et al.^6^).

A comprehensive numerical parametric study^6^ was carried out to investigate the effect of pile geometrical and soil geotechnical parameters on the ultimate capacity and settlement of LDBP. This study was based on the field measurements of the loaded to failure Alzey LDBP test. Factors affecting the response of large diameter bored piles in clayey soils were classified in this study into two main categories, as demonstrated in Fig. 4. The procedure followed in this parametric study aimed to explore the characteristic effect of each geometrical or geotechnical factor affecting the behavior of LDBP. Therefore, a particular sequence was followed in this study, as each factor was explored separately. Measurements of the reference Alzey bridge case were used to assess the variation in both pile settlement and ultimate capacity due to the change in the factor under investigation, and the influences of the other parameters were filtered out at this step. In that way, it was possible to examine the specific effect of each factor.

Results of the twenty-eight models performed were compared with the calibrated model results, and the variation in the LDBP behavior was discussed due to the change in any of the hyperparameters. Accordingly, three primary characteristics were identified to diagnose the failure of LDBP. The failure of LDBP was diagnosed by the ratio between the inducted settlement at the failure load (S_f_) and the settlement (S_f−1_) at the pre-last load increment (90% of the ultimate load). Also, it was identified through the size and the height of the plastic bulb formed around and below the base of LDBP at the failure state. In addition to that, the achievement of full friction mobilization can be ensured using Mohr–Coulomb failure criteria (σ_h_ tan Ø_i_ + c_i_).

## Hypothesis

**Figure 5:**
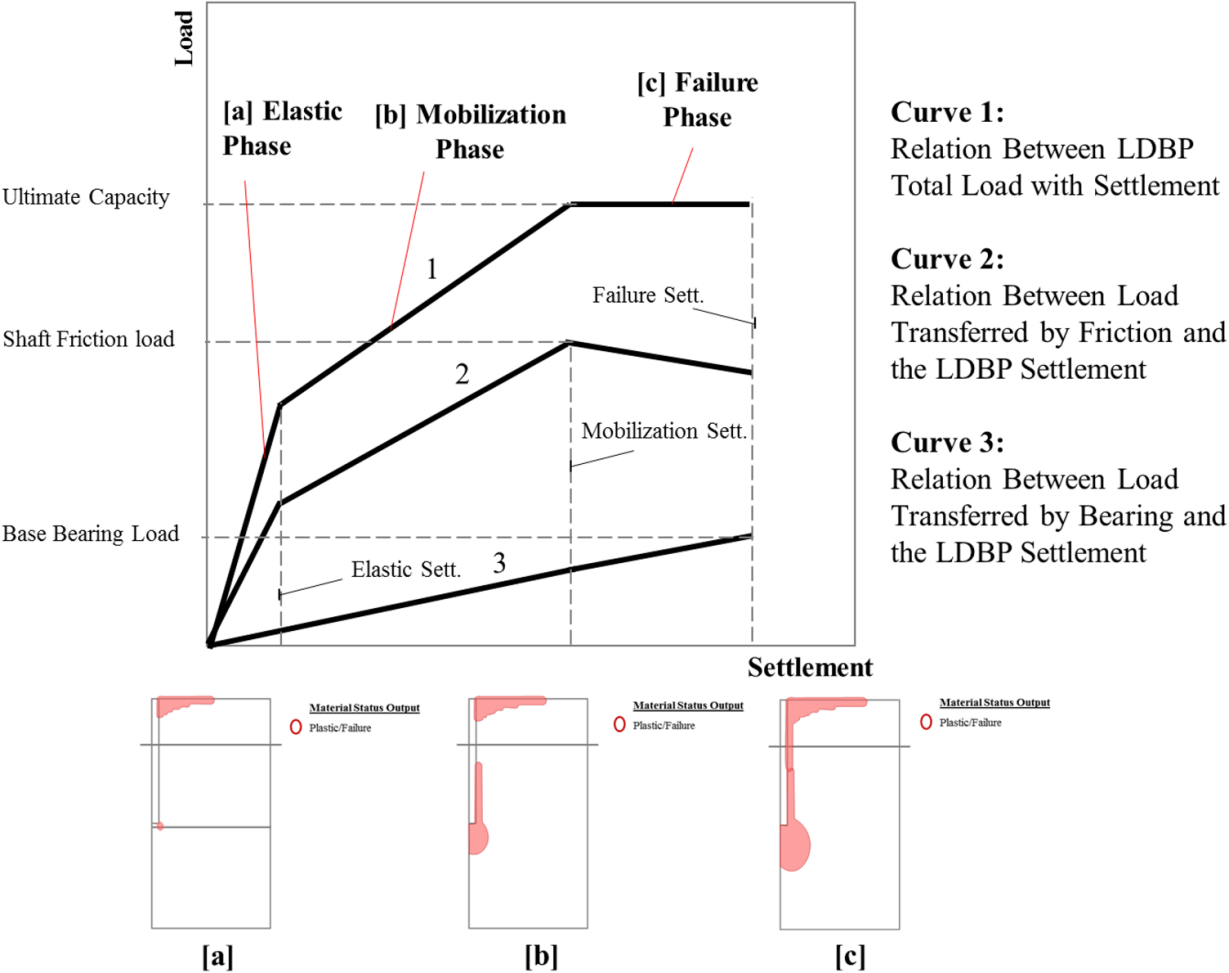
Schematic diagram of three load transfer phases describing the failure mechanism of the LDBP.

The following hypothesis is proposed depending on the conclusions of the three reference studies briefed in the previous section. Three main phases are embraced to describe the behavior of the large-diameter bored piles (LDBP). Elastic, mobilization, and failure are the three phases governing both load transfer and failure mechanisms of LDBP, as demonstrated in Fig. 5. Those three phases are expressed to organize the relation between the pile bearing and friction resistances and the expected settlement at each phase, which is essential for developing the aspired analytical approach. This three-phase concept is based on the propagation of the plastic zone formed around the LDBP’s base at different loading levels, as shown in Fig. 5a–c.

In the first stage (Elastic Phase), the interaction between soil and pile is close to being linear elastic. The applied load is expected to be predominantly transferred by friction in this stage. The first plastic points are anticipated at the base’s corners (See Fig. 5a) by the end of this elastic phase. Moving to the second phase, an evident increase in the pile settlement rate was always noticed in the mobilization phase in both field measurements and numerical results. This is attributed to the expected increase in the percentage of the load transferred by the bearing; also, soil skin friction is expected to increase and achieve its peak value of the soil shear strength at the end of the mobilization phase. As a result, plastic zones are formed at the pile base and vertically extended to a large part of the shaft, as shown in Fig. 5b. In the failure phase, the applied load is predominantly transferred by bearing, and the pile load transferred by friction tends to be constant or slightly decreased. Apparent failure is observed through the large induced pile settlement at the end of this last stage. The plastic points are extended around the base and cover almost the whole length of the pile shaft, as described in Fig. 5c.

On the other hand, as mentioned before, Meyerhof’s capacity-based method^31^ overestimates the ultimate capacity of the LDBP; in contrast, different codes’ settlement-based criteria underestimate the LDBP's ultimate capacity. This conviction was built on the results of several comparative analyses performed to assess the associated uncertainties with different capacity-based methods and settlement-based approaches recommended by various international standards^28,29,34^ for different pile loading tests with various large diameters and in different subsurfaces. That’s why Meyerhof^31^ method has been chosen for the capacity-based part of this investigation. However, a detailed assessment study will be necessary to stand on the specific etiologies behind this method's overestimated capacities for the LDBP cases.

## Methodology

The proposed analytical approach in this study was developed through three stages. First, an assessment study was performed to evaluate the reliability of the estimated LDBP ultimate capacity using the capacity-based method^31^ and the different settlement-based methods^1,8,9^. This assessment study was based on the results of the parametric numerical models of the comprehensive study^6^ that investigated the effect of different parameters on the ultimate capacity of LDBP. The parameters influencing the ultimate capacity and settlement of the LDBP were classified into two main categories. The first category included pile geometry factors: pile diameter and length. The second category was concerned with the cohesive soil parameters: effective cohesion, effective friction angle, lateral earth pressure coefficient, Young's modulus, and dilatancy angle. Consequently, the results of twenty-eight models were compared with those obtained using Meyerhof's and settlement-based methods.

The second stage was mainly concerned with the adaptations. The essential modifications were elaborated to Meyerhof’s classic formula to estimate a more reliable value for the ultimate capacity of the large diameter bored pile, based on the assessment study findings (First stage). Correlation analysis has been performed to understand the relation between load transferred and the induced settlement at the failure stage of LDBP. The relation between the plastic zone size and the bearing stress at the pile base was used to review and adopt Meyerhof's bearing capacity factors (N_c_, N_q_, and N_ɣ_). The pile head settlement to diameter ratio (S_max_/d)%, which corresponded to the maximum test load (P_max_), was also investigated to define the LDBP failure with the presence of variation in any pile geometrical or soil geotechnical parameter. On the other side, the skin friction distribution along the shafts of the twenty-eight models has also been investigated to review and adapt Meyerhof's ultimate skin friction formula.

In the third phase, the results of the parametric study^6^ were utilized to investigate the relation between soil resistance and pile settlement at the three load transfer stages (Elastic, Mobilization, and Failure). The propagation of the plastic zones around the pile shaft and under its base have been monitored to differentiate between the elastic, mobilization, and failure stages. This was vital to determine the proper factor safety that guarantees the safety and reliability of the estimated allowable capacity of the LDBP. Finally, results of the 28 numerical models with different diameters and lengths were utilized to assess the calculated ultimate friction resistance, bearing resistance, and the ultimate capacity using the modified Meyerhof analytical approach. Also, several field LDBP loading tests were employed to assess the accuracy of the developed method.

## Assessment of estimated capacity and settlement using different methods

**Figure 6:**
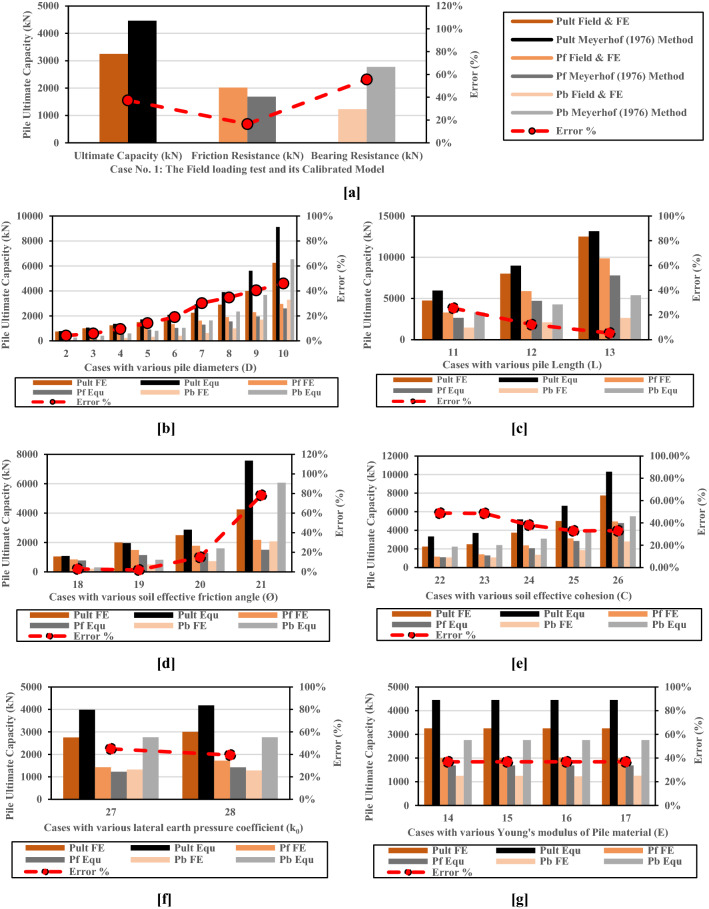
Comparison between numerical results and the ultimate pile capacity calculated using Meyerhof’s capacity-based method. (**a**) Case No. 1: The Field loading test and its Calibrated Model. (**b**) Cases with various pile diameters (D) (**c**) Cases with various pile Length (L) (**d**) Cases with various soil effective friction angle (Ø) (**e**) Cases with various soil effective cohesion (C) (**f**) Cases with various lateral earth pressure coefficient (k_0_) (**g**) Cases with various Young's modulus of Pile material (E).

Table 1 summarizes the results of the twenty-eight numerical models and the estimated ultimate capacity of the LDBP cases using Meyerhof's^31^ capacity-based method. Figure 6 compares the obtained ultimate pile capacity using finite element analysis and Meyerhof’s formula (Eq. 1) and shows the error percentage of each case.

**Table 1:** Results of twenty-eight numerical models for piles with various characteristics and mechanical properties of soil.

Case no	Input								R	Results obtained from numerical analyses					Results calculated using the meyerhof method (1976)			Ult. load
	D	L	C	Ø	K_0_	γ	ψ	E		P_ult_ FE	P_f_ FE	P_b_ FE	Sett FE	S/D%	P_ult_ Equ	P_f_ Equ	P_b_ Equ	Error %
Units	(m)	(m)	(kN/m^2^)	0	–	(kN/m^2^)	O	(kN/m^2^)		(kN)	(kN)	(kN)	(mm)	%	(kN)	(kN)	(kN)	%
1 (The calibrated model)	1.3	9.5	20	22.5	0.8	20	0.1	45000	1	3250	2019.58	1230.42	70.24	5.40	4446.27	1687.08	2774.19	37.27
2	0.4	9.5	20	22.5	0.8	20	0.1	45,000	1	750	666.79	83.21	17.98	4.5	781.75	519.102	262.64	4.23
3	0.5	9.5	20	22.5	0.8	20	0.1	45,000	1	1000	876.89	123.11	29.91	5.98	1059.26	648.877	410.38	5.93
4	0.6	9.5	20	22.5	0.8	20	0.1	45,000	1	1250	1020.7	229.3	40.28	6.71	1369.61	778.653	590.95	9.57
5	0.7	9.5	20	22.5	0.8	20	0.1	45,000	1	1500	1179.06	320.94	47.05	6.72	1712.8	908.428	804.35	14.19
6	0.8	9.5	20	22.5	0.8	20	0.1	45,000	1	1750	1337.73	412.27	47.15	5.89	2083.2	1038.2	1044.99	19.04
7	1	9.5	20	22.5	0.8	20	0.1	45,000	1	2250	1620.64	629.36	54.26	5.43	2930.55	1297.75	1632.8	30.25
8	1.2	9.5	20	22.5	0.8	20	0.1	45,000	1	2900	1905.81	994.19	61.81	5.15	3908.54	1557.31	2351.23	34.78
9	1.5	9.5	20	22.5	0.8	20	0.1	45,000	1	4000	2297.37	1702.63	96.23	6.42	5620.43	1946.63	3673.8	40.51
10	2	9.5	20	22.5	0.8	20	0.1	45,000	1	6250	2958.3	3291.7	133.3	6.67	9126.71	2595.51	6531.2	46.03
11	1.3	13	20	22.5	0.8	20	0.1	45,000	1	4750	3288.27	1461.73	87.23	6.71	5961.08	2644.45	3316.63	25.50
12	1.3	19	20	22.5	0.8	20	0.1	45,000	1	8000	5888.34	2111.66	137.32	10.56	8978.17	4706.36	4271.81	12.23
13	1.3	26	20	22.5	0.8	20	0.1	45,000	1	12,500	9864.6	2635.4	160.92	12.38	13,169.7	7783.54	5386.2	5.36
14	1.3	9.5	20	22.5	0.8	20	0.1	20,000	1	3250	2003.27	1246.73	155.58	11.97	4446.51	1687.08	2759.43	36.82
15	1.3	9.5	20	22.5	0.8	20	0.1	30,000	1	3250	2004.19	1245.81	103.76	7.98	4446.51	1687.08	2759.43	36.82
16	1.3	9.5	20	22.5	0.8	20	0.1	60,000	1	3250	2021.24	1228.76	52.94	4.07	4446.51	1687.08	2759.43	36.82
17	1.3	9.5	20	22.5	0.8	20	0.1	80,000	1	3250	1994.13	1255.87	39.67	3.05	4446.51	1687.08	2759.43	36.82
18	1.3	9.5	20	0	1	20	0.1	45,000	1	1050	837.9	212.1	9.96	0.77	1080.71	775.58	305.13	2.92
19	1.3	9.5	20	10	0.82	20	0.1	45,000	1	2000	1494.34	505.66	30.71	2.36	1961.05	1142.70	818.35	1.95
20	1.3	9.5	20	15	0.74	20	0.1	45,000	1	2500	1773.95	726.05	41.63	3.20	2873.15	1275.38	1597.77	14.93
21	1.3	9.5	20	30	0.5	20	0.1	45,000	1	4250	2173.79	2076.21	132.08	10.16	7573.06	1503.22	6069.83	78.19
22	1.3	9.5	5	22.5	0.8	20	0.1	45,000	1	2250	1167.21	1082.79	72.7	5.59	3347.43	1105.4	2242.04	48.77
23	1.3	9.5	10	22.5	0.8	20	0.1	45,000	1	2500	1417.04	1082.96	68.1	5.24	3713.79	1299.29	2414.5	48.55
24	1.3	9.5	30	22.5	0.8	20	0.1	45,000	1	3750	2380.05	1369.95	74.54	5.73	5179.23	2074.87	3104.36	38.11
25	1.3	9.5	50	22.5	0.8	20	0.1	45,000	1	5000	3135.2	1864.8	103.16	7.94	6644.67	2850.45	3794.22	32.89
26	1.3	9.5	100	22.5	0.8	20	0.1	45,000	1	7750	4954.8	2804.2	119.34	9.18	10,308.3	4789.4	5518.86	33.01
27	1.3	9.5	20	22.5	0.43	20	0.1	45,000	1	2750	1426	1323.34	91.45	7.03	3983.97	1224.53	2759.43	44.87
28	1.3	9.5	20	22.5	0.62	20	0.1	45,000	1	3000	1719.03	1280.97	85.52	6.58	4182.34	1422.91	2759.43	39.41

Where D: pile diameter, L: pile length, E_elastic_: Young's modulus of Pile material, Ø′: soil effective friction angle, c′: soil effective cohesion, K_0_: lateral earth pressure coefficient, ψ: dilatancy angle, R: interface strength reduction factor.

Results of the calibrated numerical model of the loaded to failure LDBP test (1.30 m diameter) were compared with those calculated using Meyerhof's method. As observed from Fig. 6a, the estimated ultimate LDBP capacity using this classical method was 37% greater than the one measured in the field loading test and its calibrated numerical model. Figure 6 also indicates that Meyerhof's method almost overestimated the ultimate capacities of the large diameter bored piles of all cases with diameters greater than 60 cm, and the error percentage increased with the pile diameter increases, as shown in Fig. 6b. The error percentages ranged from 14 to 46% in the cases with large diameters (greater than 60 cm), which is relatively high compared with the small diameter cases (Cases 2 and 3); as the error percentages ranged from 4 to 6% in those small diameter cases. Conversely, the error percentages were linearly decreased with the pile length increases, as demonstrated in the results of cases 11–13 (Fig. 6c). In the presence of variation in the mechanical properties, the error percentages increased with friction angle increases (Fig. 6d). In contrast, it decreased with soil cohesion, and the lateral earth pressure coefficient increase (Fig. 6e–f). In addition, soil young’s modulus did not affect the error percentage as it was not included in Meyerhof’s capacity-based formula (Fig. 6g).

**Figure 7:**
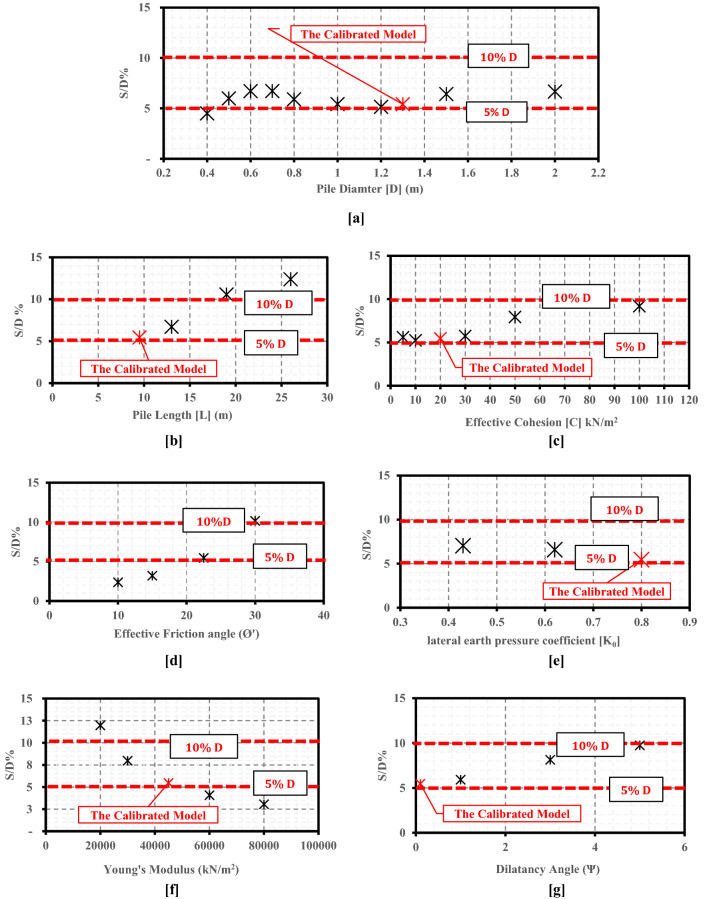
Variation in the pile's settlement to diameter percentage (S/D%) due to the change in different parameters. (**a**) Pile diameter (D). (**b**) Pile length (L). (**c**) Soil effective cohesion (c′). (**d**) Soil effective friction angle (Ø′). (**e**) Soil lateral earth pressure coefficient (k_0_). (**f**) Soil Young’s modulus (E_s_). (**g**) Soil dilatancy angle (Ѱ).

On the other side, the pile's settlement to diameter percentage (S/D%) was also calculated using the results of the 28 numerical models (Table 1). The relations between pile geometrical parameters, soil geotechnical parameters, and the (S/D%) are presented in Fig. 7. It can be seen from Fig. 7a that pile with a diameter of 0.40 m (Small Diameter pile) achieved a settlement with a percentage of 4.50% of its diameter (S/D%). This percentage increased to near-equal to 7.0% (6.71%) when the pile diameter was increased to 0.60 m. However, the S/D% percentage was obtained as 6.71% of the pile No.4 diameter (0.70 m), which almost equals the same value obtained for the pile with a diameter of 0.60 m; in spite of the corresponding increase in ultimate capacity due to diameter increase (Q_ult_ increases from 1250 kN for pile with diameter 0.60 m to be 1500 kN for the pile with diameter 0.70 m). Fundamental to note that despite the significant increase of the ultimate load at the last large diameter pile case (2.0 m diameter), but the percentage (S/D%) at this case (6.67%) didn't exceed the obtained percentage at the third small diameter pile case (0.60 m diameter) which was 6.71%, however, the higher obtained ultimate capacity (6250 kN) of the tenth pile with a diameter of 2.00 m. This result highlights the main advantages of the large-diameter bored piles as they are employed most frequently to support heavy loads and minimize settlement^1^.

Figure 7a,c,d, and g demonstrate that the calculated pile settlement percentages (S/D %) increase with increases in pile length (L), clay effective cohesion (c′), effective friction angle (Ø′), or soil dilatancy angle (Ѱ). Conversely, as shown in Fig. 7e and f, the S/D percentage decreased with the increases in lateral earth pressure coefficient (K_0_), and soil Young's modulus (E). Those results revealed that the obtained S/D% percentage ranges from about 5–12% of pile diameter. Besides, it is affected by both pile geometry and soil geotechnical parameters, which disagrees with the different settlement-based methods^1,8,9^.

## Adaptation of Meyerhof’s capacity-based method (1976)

Based on the assessment results presented in the previous section, the obtained bearing resistance using the Meyerhof method was more significant than the field measured value of the loaded to failure LDBP test (Case 1, Table 1). The field measured bearing resistance represented about 45% of the ultimate bearing resistance calculated using Meyerhof’s classic formula. Therefore, the LDBP’s bearing resistance will be the starting point in this adaptation phase.

### Ultimate bearing resistance of the LDBP

**Figure 8:**
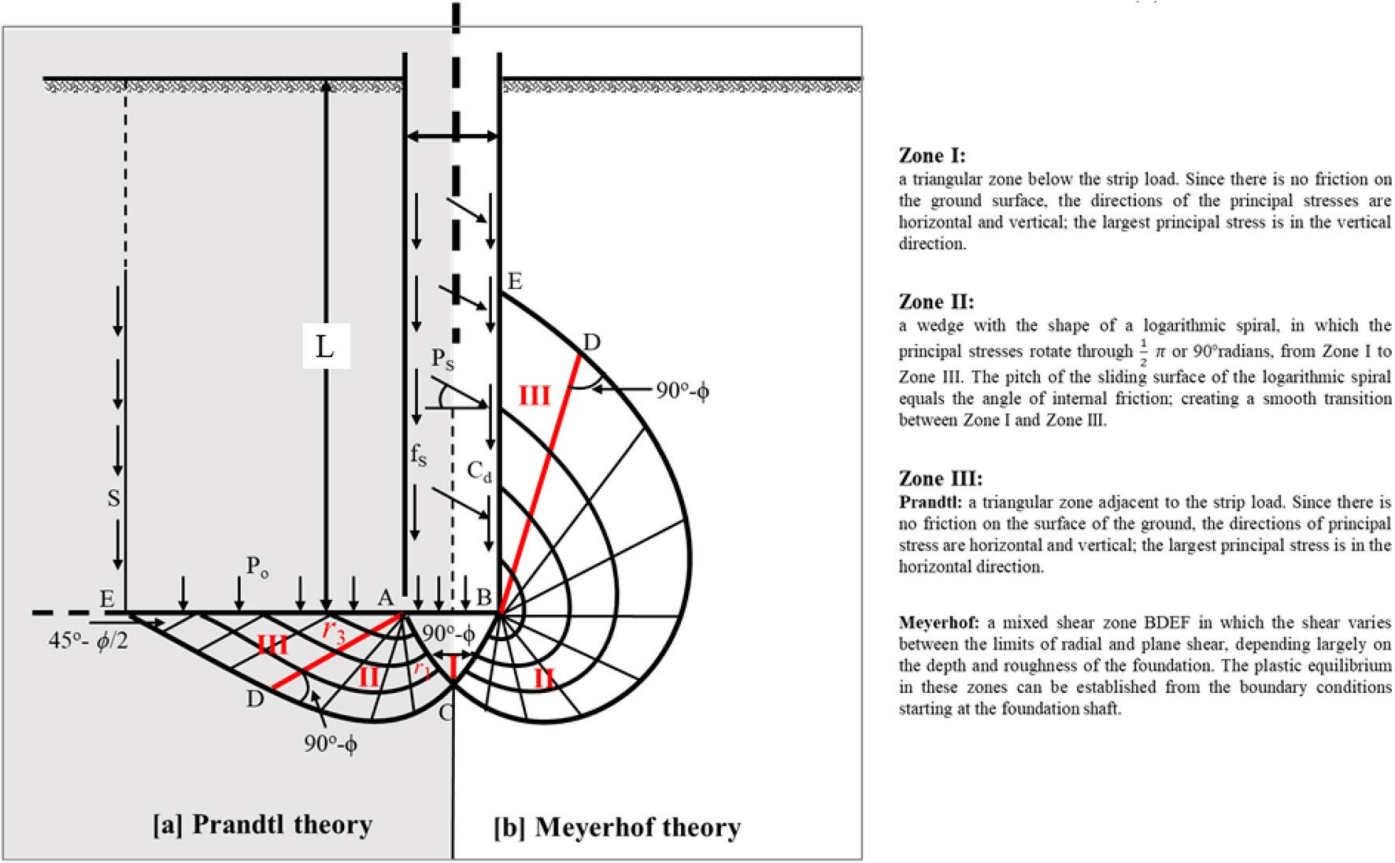
Plastic zones near the rough pile foundation.

Prandtl^39^, Reissner^40^, Terzaghi^41^, Meyerhof^30^ and Vesic^42^ have studied the bearing capacity of shallow and deep foundations. They all built their theories based on plasticity wedge failure mechanism but with different shear patterns and rupture lines reverting to the shaft. Most of those studies assumed a theoretical distribution of the contact pressure on the base of a foundation to approximately determine the ultimate bearing capacity from the corresponding distribution of the passive earth pressure on the central zone of material below the base (Fig. 8, and Eq. (2)). Accordingly, different derivations were conducted to estimate the bearing capacity factor N_q_.

2 $$ {\text{p}} = {\text{qN}}_{{\text{q}}} $$

In particular, Meyerhof determined the bearing capacity factor (N_q_) based on the passive earth's normal and tangential components along with the AC plane (Fig. 8), as expressed in Eq. (3). Besides, Meyerhof^30^ stated that at great foundation depths (e.g., long piles), the N_c_ and N_ɣ_ components could be ignored compared to the great value of the N_q_ component. Also, as given in Eqs. (4) and (5), the values of N_c_ and N_ɣ_ are governed by the value of the N_q_ factor.

3 $$ {\text{N}}_{{\text{q}}} = {\text{e}}^{{\uppi \tan\upphi }} \tan^{2} \left( {45 +\upphi /2} \right) $$

4 $$ {\text{N}}_{{\text{c}}} = \cot\upphi \left( {{\text{N}}_{{\text{q}}} - 1} \right) $$

5 $$ {\text{N}}_{\upgamma } = \left( {{\text{N}}_{{\text{q}}} - 1} \right)\tan \left( {1.4\upphi } \right) $$

Meyerhof^31^ developed his chart of the bearing capacity factors N_c_, N_q,_ and N_ɣ_ to obtain the point bearing resistance of the bored piles_._ In this chart, the bearing capacity factors were dependent not only on the soil friction angle (Ø) but also on the depth and shape of the foundation and the depth of the overlying soil layer. Thus, factor (N_q_) was predominantly affected by the soil friction angle (Ø) and lateral earth pressure coefficient (K) at the pile base. Therefore, the passive lateral earth pressure coefficient (K_p_) was considered in this derivation (Eq. 3).

**Figure 9:**
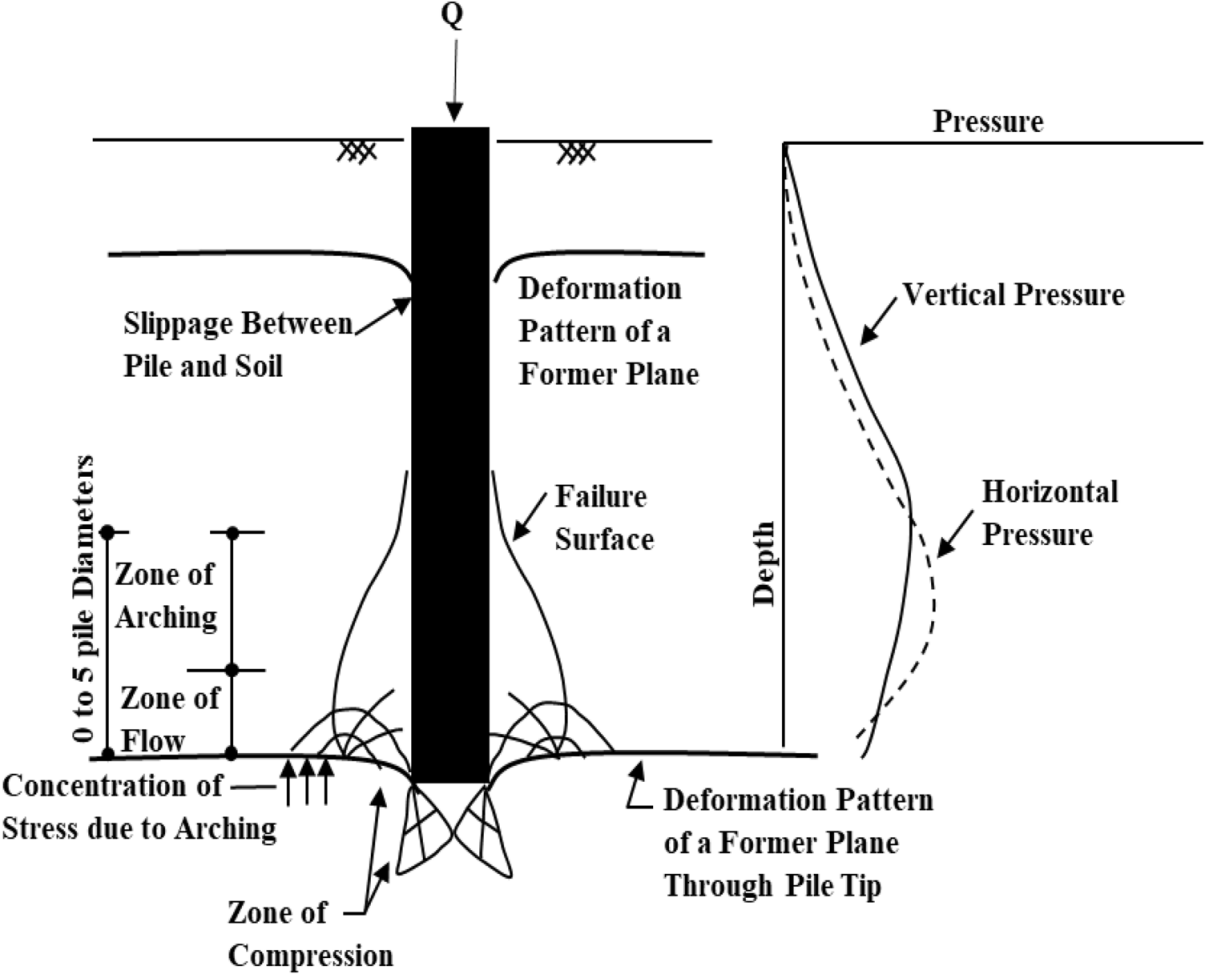
Change in the stresses around the pile and at the tip due to arching action^44^.

Meyerhof's analytical solutions^30,31^ ignored the effect of arching action that occurs around the pile’s base at the failure state. With that in mind, it was proven in several studies^37,43–45^ that this arching action is affecting the vertical and horizontal stresses around the pile’s shaft and base. As explained in Fig. 9, after full mobilization, at a distance of about (H/3) above the base, the horizontal soil stresses were observed to be greater than the vertical soil stresses. Also, both vertical and horizontal stresses were accordingly decreased at the pile base level after the full mobilization. This was also consistent with the calibrated model results^37^, as shown in the results presented in Fig. 3b.

**Figure 10:**
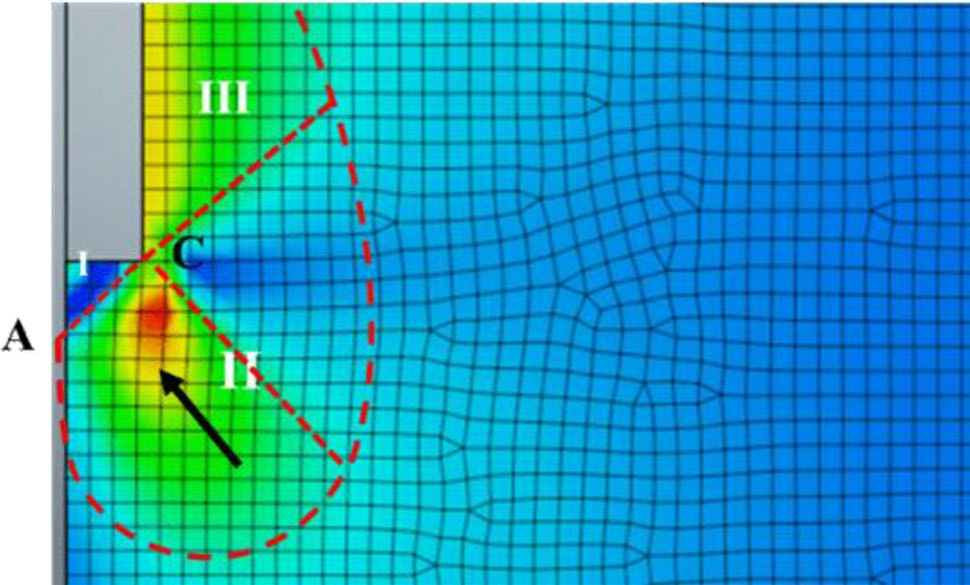
Shear stress distribution around the LDBP base at the failure state.

In the presence of the arching action and the change in soil compressibility at the failure state, the acting pressure on triangular wedge AC might no longer be a fully passive pressure in all cases. The results of vertical and horizontal stresses around the Alzey Bridge LDBP model (Fig. 10) base were determined at the failure load using the calibrated numerical model^37^. The lateral earth pressure coefficient (K_Failure_) was determined as $\left( {\frac{\sigma h}{{\sigma v}}} \right)$. By comparing the obtained K_Failure_ with the passive lateral earth pressure coefficient (K_P_) determined using the Rankine formula^46^ (Eq. 6), it was found that K_Failure_ represents 82% of the entire K_P_. This may explain the great N_q_ values and, hence, Meyerhof's method overestimated bearing resistances.

6 $$ {\text{K}}_{p} = \frac{1 + \sin \emptyset }{{1 - \sin \emptyset }} = \tan^{2} \left( {45 +\upphi /2} \right) $$

Similarly, the models of the comprehensive parametric study^6^ were utilized to investigate the change in K_Failure_ with the change of the different geotechnical parameters. The results of vertical and horizontal stresses around the pile base were obtained at the failure load for the 28 numerical models. For instance, five models with different friction angles (0°, 10°, 15°, 22.5°, and 30°) are presented in Table 2.

**Table 2:** The ratio between vertical and horizontal stresses around pile base and under failure load for different effective friction angle values.

Ø′	The ratio between horizontal and vertical stresses around the pile base under failure load (K)	Passive lateral earth pressure coefficient (K_P_) using rankine formula	K_Failure_/K_P_
0	1.0	1.0	1.00
10	1.42	1.42	1.00
15	1.59	1.69	0.94
22.5	1.83	2.24	0.82
30	2.08	3	0.69

For the first two cases with small soil effective friction angles of 0° and 10°, Table 2 shows that the obtained lateral earth coefficient around the pile base (K_Failure_) using the numerical models equals the passive earth pressure coefficient (k_p_) calculated using the Rankine equation. However, the difference between the obtained K_Failure_ and K_p_ was increased with effective friction angle increases. For instance, the obtained lateral earth pressure coefficient (K_Failure_) was about 70% of the passive earth pressure coefficient (k_p_) with a 30° friction angle.

**Figure 11:**
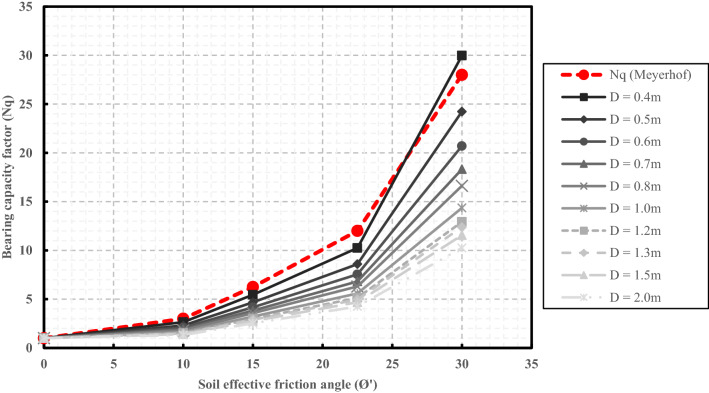
Comparison between Meyerhof ^30^ values for the N_q_ bearing capacity factor and the ones obtained using numerical analysis.

On the other hand, the plastic zone formation sequence around the pile at the failure state has also been explored through the parametric numerical study^6^. Based on the twenty-eight model's results, It was found that the plastic bulb was obviously increased in size and height not only with soil friction angle increases but also with the pile diameter increases. For instance, the diameter of the plastic blub (D_p_) under the base of the first pile case with a diameter of 0.40 m was 2.14 m (~ 5D), while the greatest size of the plastic bulb was achieved as 7.08 m (3.54D) when pile diameter increases to be 2.00 m. However, almost the same diameter of the plastic bulb is obtained in all cases with equal diameters and different lengths. Those findings are consistent with^44^ conclusions (Fig. 9), and the zone of arching action was located around the pile’s base in a zone ranging from 0 to 5 times the pile’s diameter, according to the change in soil effective friction angle and the pile’s diameter. Thus, the parametric numerical models with different diameters were utilized to calculate the N_q_ factor with the same Meyerhof^30^ formula (Eq. 3), but with determined K _Failure,_ instead of the K_p_. The obtained N_q_ values were compared with Meyerhof's ones in Fig. 11.

It can be seen from Fig. 11 that good agreement between the Meyerhof’’s and the calculated N_q_ values using numerical results was only obtained for cases with friction angles less than 15° In addition, very good agreement was found for the cases with diameters less than 60 cm. However, the difference between obtained N_q_ values and Meyerhof’s values increased with pile diameter increases. At friction angles higher than 15°, Meyerhof’s suggested values were more significant than those obtained from numerical analyses. The difference between obtained N_q_ values and Meyerhof values was about 46% in the last case with a friction angle of 30°. The adapted N_q_ values addressed in Fig. 11 will be used for validation purposes in the coming sections of this study.

### Ultimate friction resistance of the LDBP

Fundamental to state that the difference between the estimated skin friction using Meyerhof^31^ and the field measurements was not as big as the difference noted in bearing stress results, as presented before in “Methodology” section. According to Meyerhof^31^, the unit of frictional resistance along pile length increases with pile depth, to be with a maximum value at a depth of half pile length, and remains constant after that. So that Meyerhof defined half pile length (L/2) as a critical depth. With that in mind, critical depth is one of the most debatable issues in soil–pile interaction studies. Therefore, to ensure its existence, the distribution of unit skin friction along pile length was investigated through the four cases with different lengths ranging from 9.50 to 26 m (1, and 11–13).

**Figure 12:**
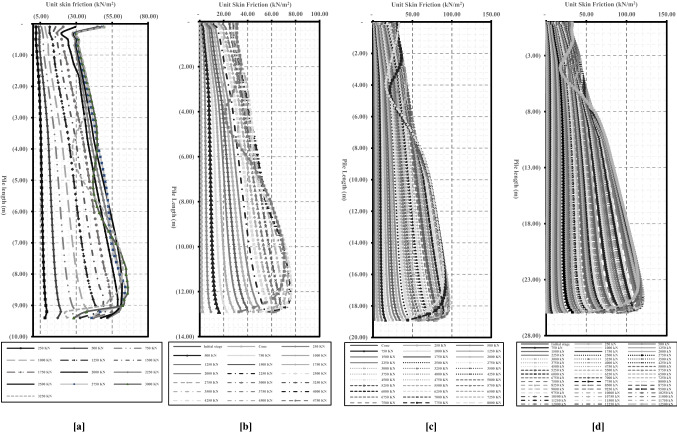
Results of unit skin friction distribution with depth obtained using four numerical models with different lengths. (**a**) Length of 9.50 m. (**b**) Length of 13.0 m. (**c**) Length of 19.0 m. (**d**) Length of 26.0 m.

As shown in Fig. 12, numerical results of the distribution of the unit skin friction along pile length indicate that the unit skin friction was increased from the pile head level to the level near the pile base and only decreased at the pile base level after full mobilization. The decrease in skin friction around the pile base was attributed to the arching action effect, as explained before in Figs. 3b and 9. This response was consistent in the four pile cases with various lengths, which pinpoint that, there is no existence for the critical depth in this study, and unit friction is increased with loading and with depth increases to achieve its maximum value that equals the soil shear strength (Mohr–Coulomb failure criteria (σ_h_ tan Ø_i_ + c_i_) at the failure load of the pile.

**Figure 13:**
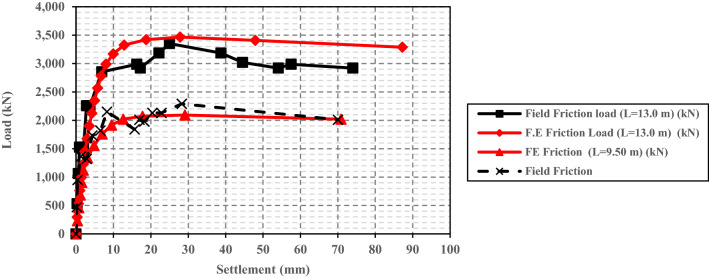
The relation between the obtained average unit skin friction and settlement for the four numerical models with different lengths.

Fundamental to note that the results of the two models with lengths of 9.50 and 13.0 m were compared with the provided field measurements of the average unit skin friction in the case study^36^. Figure 13 presents the relation between the obtained pile friction resistance and settlement of the two numerical models with different lengths. As shown, good agreement was obtained between the numerical results and field measurements of the two loading tests.

Furthermore, the impact of clay effective cohesion (C′), effective friction angle (Ø′), pile length (L), and lateral earth pressure (K_0_) were investigated in the parametric reference study^6^. It was found that whatever the change in each of those parameters values, the full friction mobilization always occurs when the transferred shear stress from the large diameter pile to the surrounding soil achieves the value of the soil shear strength according to Coulomb-Mohr’s theory of failure (c_a_ + Kσ_v_ tan δ).

Ezzat et al.^37^ also highlighted that at the initial stage, the value of the lateral earth pressure coefficient equals the at-rest value (K_0_) (See Fig. 3a). Then, the lateral earth pressure coefficient (K) increased with loading increases during pile loading. Also, it was observed from field tests and numerical studies that at the failure state, lateral earth pressure coefficient (K) was not a constant value along the pile length, but it changed from its most substantial value at the pile head level to its smallest value at the pile base (See Fig. 3c), due to change in both vertical and horizontal stresses corresponding to the arching action occurs at failure state as explained by Franke^44^, Reese et al.^47^ and Kamal et al.^45^. With that in mind, as shown in Fig. 3c, at the pile head level, the lateral earth pressure coefficient (K) was higher than the initial lateral earth pressure coefficient (K_0_). At the middle levels of the pile, the k value was near equal unity. In contrast, at the pile base level, the lateral earth pressure coefficient (K) was the same or less than the initial lateral earth pressure coefficient (K_0_). This finding also agrees with Rollins et al.'s^48^ conclusions.

**Figure 14:**
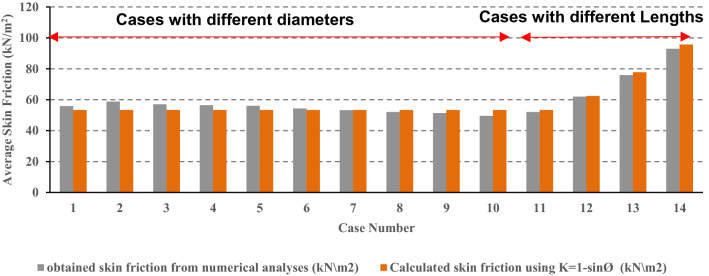
Comparison between the obtained average skin friction using fourteen numerical models with different geometries and the calculated ones using at-rest lateral earth pressure coefficient (K_0_).

To simplify the analysis, and based on the results of the parametric study; it was found that at the ultimate state, the average value of the lateral earth pressure coefficient (K_avg_) along the pile length was usually near equal to the initial (at rest) lateral earth pressure coefficient (K_0_). As shown in the cases sample presented in Fig. 14, the average skin friction obtained using fourteen (14) numerical models for piles with different geometries were compared with those calculated using at-rest lateral earth pressure coefficient (K_0_) and good agreement was noted.

Consequently, it may be wiser to determine the average unit skin friction using the average lateral earth pressure coefficient (K_avg_) instead of average length (L/2). Thus, the average ultimate skin friction (Drained condition) could be modified as in Eq. (7);

7 $$ f_{su} = {\text{c}}_{{\text{a}}} + {\text{K}}_{{{\text{avg}}}}\upgamma \,\,{\text{L}}_{{\text{e}}} \tan\updelta . $$

where L_e_ is effective pile length which could be defined as the full pile length after deducting the length of the affected zone by the arching action at the pile base, as explained before, the affected zone length ranges from zero to five times the pile diameter, according to the soil type^15,37,43–45^.

### Modified Meyerhof capacity based method

Previous sections presented a detailed assessment and adaptation of the Meyerhof^31^ formula for estimating the ultimate capacity of the large diameter bored piles. The derivations and the assumptions considered in this method were reviewed, and the responsible factors that led to the overestimated LDBP ultimate capacity values have been identified. Accordingly, Meyerhof's^31^ formula could be modified as given in the modified Eq. (8); for the soil that gives both adhesion and friction angle (Drained Condition).

8 $$ {\text{P}}_{{{\text{ult}}}} = {\text{A}}_{{\text{s}}} \left( {{\text{c}}_{{\text{a}}} + {\text{K}}_{{{\text{avg}}}}\upgamma {\text{L}}_{{\text{e}}} \tan\updelta } \right) + {\text{A}}_{{\text{b}}} \left( {{\text{CN}}_{{\text{c}}}^{*} +\upgamma {\text{LN}}_{{\text{q}}}^{*} +\upgamma \frac{{\text{D}}}{2}{\text{N}}_{\upgamma }^{*} } \right) $$

where γ: Soil unit weight (kN/m^2^); A_s_: Pile surface area (m^2^); A_b_: Pile cross section area (m^2^); D: Pile diameter (m). L: Pile length (m); L: Pile effective length (m); ϕ′: clay soil effective friction angle (°); C: clay soil cohesion (kN/m^2^). δ: angle of friction of the soil on the shaft; C_a_: the adhesion per unit area (kN/m^2^), K_avg_: The at-rest lateral earth pressure coefficient; N_c_^*^, N_q_^*^, and N_γ_^*^: the modified bearing capacity factors. For drained conditions, it is recommended to obtain the N_q_^*^ factor from Fig. 11 based on soil effective friction angle (ϕ′). The two factors N_c_^*^ and N_γ_^*^ should be calculated using Eqs. (4, and 5) using the modified N_q_* factor.

### Verification of the modified Meyerhof approach

**Figure 15:**
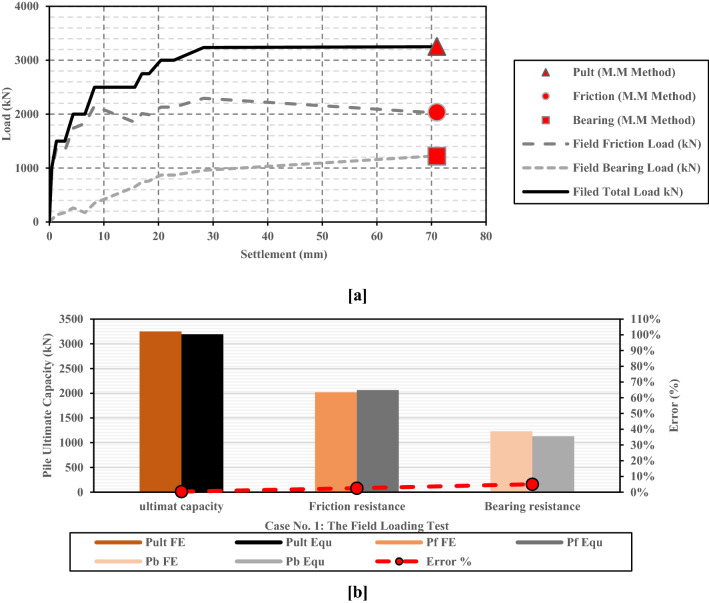
Comparison between the calculated pile friction resistance, bearing resistance, and the ultimate capacity using the modified approach and the in-situ measurements of the case history^36^. (**a**) Load-settlement relationship (**b**) Ultimate pile resistances.

The large-diameter bored pile of the Alzey Bridge case study was utilized as an example to evaluate the accuracy of the modified approach. As presented before, the ultimate capacity of this large-diameter pile (1.30 m diameter) was 3250 kN. The ultimate capacity of the LDBP case was also calculated using the Modified Meyerhof method (Eq. 8). Thus, results calculated using the modified approach were compared with field measurements. As shown in Fig. 15a and b, excellent agreement was obtained; and the error percentages were 0.34%, 2.51%, and 5.02% for the ultimate friction and bearing capacities, respectively.

## Validation and discussion

**Figure 16:**
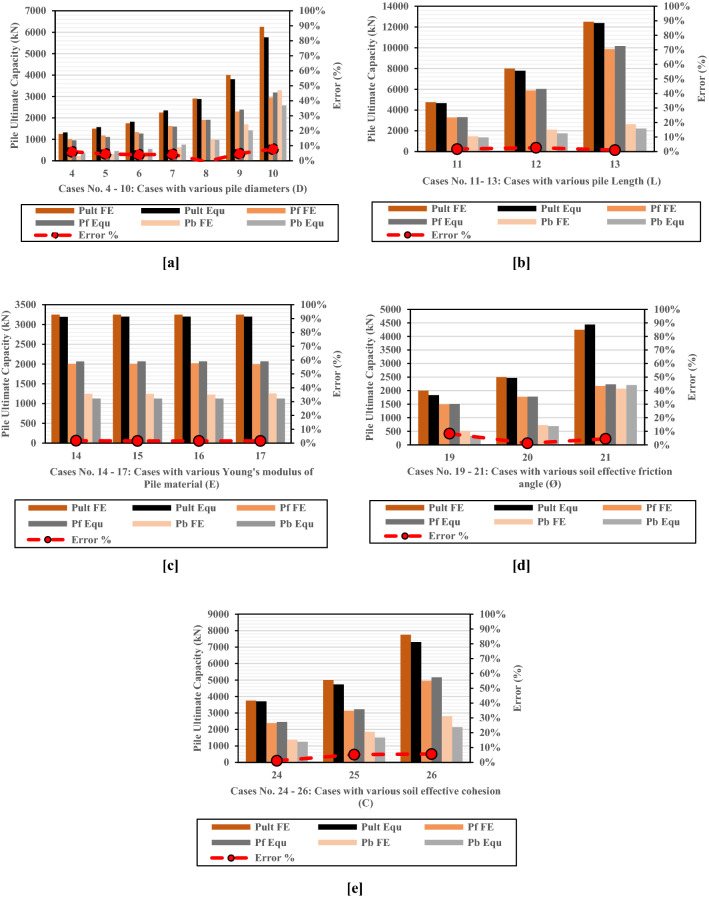
Comparison between numerical results and the LDBP ultimate pile capacity calculated using the proposed Approach. (**a**) Cases with various pile diameters (**b**) Cases with various pile Length (**c**) Cases with various Young's modulus of Pile material (**d**) Cases with various soil effective friction angle (**e**) Cases with various soil effective cohesion.

Results of twenty numerical models^20^ (Appenxis-A) for LDBPs with variable geometries and different soil parameters have been used to validate the proposed combined methods. In comparison, soil and piles parameters of those twenty cases were used to calculate the ultimate bearing, friction, and total capacities of the large diameter bored piles using the Modified Meyerhof Method (Eq. 8). Furthermore, the determined ultimate capacities using the combined approach were also allocated on the same load settlement relationships of the twenty cases (Appenxis-A). Concerning the variable geometries and different soil parameters included, the comparison has shown that the Modified Meyerhof capacity-based method could estimate accurate values for the ultimate capacity of the LDBP in both working and failure states, and the error percentages range from 0.267 to 7.75%, as shown in Fig. 16a–e.

**Figure 17:**
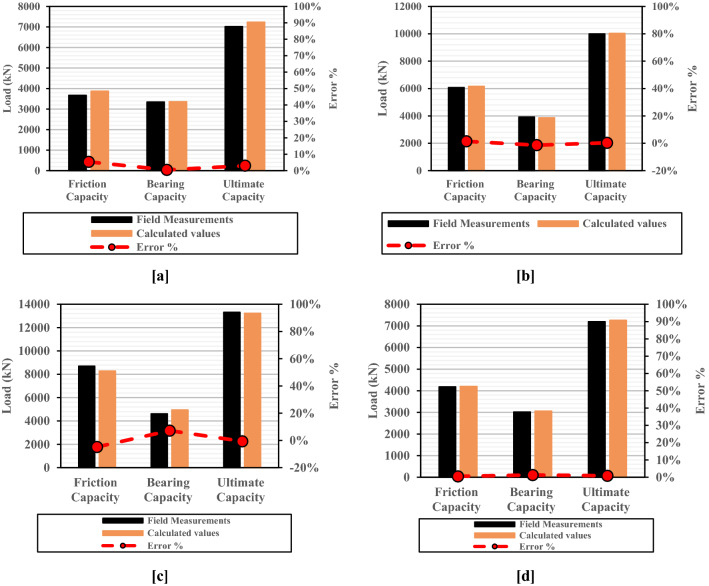
Comparison between field measurements of four insitu LDBP loading tests and the ultimate pile capacity calculated using the Modified Meyerhof method. (**a**) Case (1) for an LDBP (D = 0.7 m) installed in multi-layered soil (**b**) Case (2) for an LDBP (D = 0.8 m) installed in multi-layered soil (**c**) Case (1) for an LDBP (D = 1.0 m) installed in multi-layered soil (**d**) Case (1) for an LDBP (D = 1.2 m) installed in clayey sand soil.

In the same line, to ensure the reliability of the proposed approach, four LDBP different in-situ loading tests were utilized to evaluate the accuracy of the modified Meyerhof method. The soil and pile parameters of the four case histories are given in Table A (Appenxis-A). More details about the four case histories could be found in Zhang et al.^20^ and Hernan and Juan^49^. The comparison between the results calculated using the Modified Meyerhof capacity-based method and the field measurements of the four LDBP in-situ loading tests confirmed the reliability of the proposed method, as the error percentages range from 0.21 to 7.18%, as shown in Fig. 17a–d.

The presented results could enormously strengthen the proposed hypothesis, and the response of large diameter bored piles (LDBP) in stiff clay soil can be described through three primary stages to achieve its expected large settlement at the failure state. Those three phases are the elastic phase, the mobilization phase, and the failure phase, as described in Fig. 5.

The parametric study Al-Atroush et al.^20^ showed that the ultimate pile load represents about 1.5–2.5 times the load at which the first plastic point was formed around the pile base edge. The first plastic point formed around the pile base edge was the adopted criterial to identify the endpoint of the elastic phase. Based upon, using a factor of safety (F.S) of 2–2.50 or greater will guarantee that the compressible soil is still in its elastic phase, and the elastic soil young’s modulus can then be used to calculate the pile settlement under the working load (Q_ult_/2.5). It was also noted that the interaction between soil and LDBP in the elastic phase was close to being linear elastic. The applied load was predominantly transferred by friction in this stage (more than 90% of the total applied load). The ratios between the load transferred by both friction $\left( {\frac{Qf}{{Qw}}} \right)$ or bearing $\left( {\frac{Qb}{{Qw}}} \right)$ compared to the total load could be used to obtain the expected settlement at the elastic phase.

## Conclusions

This study introduced an analytical approach for estimating the ultimate capacity of the large diameter bored piles (LDBP). The proposed approach was developed based on an adaptation of Meyerhof’s classical (1976) method. The suggested adaptations were based on the results of the in-situ loaded to failure LDBP test and a related comprehensive parametric numerical study.

A detailed assessment study was performed to evaluate the reliability of Meyerhof’s classical formula (1976) and some common settlement-based methods in estimating the ultimate capacity of LDBP. This assessment study revealed that Meyerhof's method almost overestimated the ultimate capacities of the twenty-six large diameter bored piles cases (with a diameter greater than 60 cm). Also, the error percentage increased with the pile diameter increase. The error percentages ranged from 14 to 46% in the LDBP cases, which is relatively high compared with the small diameter cases. The error percentages ranged from 4 to 6% in those small diameter cases. On the other hand, the assessment study results showed that the calculated pile settlement percentages (S/D %) ranged from about 5–12% of pile diameter. Besides, it is affected by both pile geometry and soil geotechnical parameters, which disagrees with the different conventional settlement-based methods utilized in different international design standards.

The relatively high bearing capacity factors utilized in the bearing resistance calculation were the primary etiology behind Meyerhof’s classical formula (1976) overestimated capacities for the LDBP cases. Meyerhof determined the bearing capacity factor (N_q_) based on the passive earth pressure's normal and tangential components acting on the triangular shear plane formed under the pile base. However, the parametric numerical study results revealed that arching action occurs after the full friction mobilization affects both the vertical and horizontal stresses around the pile’s shaft and base. Thus, in the presence of the arching action and the change in soil compressibility at the failure state, the acting normal and tangential components on the triangular wedge might no longer be an entirely passive pressure in all cases.

Numerical results indicated that the unit skin friction increased from the pile head level to the level near the pile base and only decreased at the pile base level after full mobilization due to the arching action effect. This response was consistent in the four pile cases with various lengths, which pinpoint that, there is no exitance for the critical depth in this study, and unit friction is increased with loading and with depth increases to achieve its maximum value that equals the soil shear strength (Mohr–Coulomb failure criteria (σ_h_ tan Ø_i_ + c_i_) at the failure load of the pile.

Excellent agreement was obtained between the theoretically calculated friction capacity and the numerical results when the average unit skin friction was determined using the average lateral earth pressure coefficient (K_avg_) instead of average length (L/2). Furthermore, based on the results of the parametric study, it was found that at the ultimate state, the average value of the lateral earth pressure coefficient (K_avg_) along the pile length was usually near equal to the initial (at rest) lateral earth pressure coefficient (K_0_).

The response of large diameter bored piles (LDBP) in stiff clay soil can be described through three main stages to achieve its expected large settlement at the failure state. Those three phases are the elastic phase, the mobilization phase, and the failure phase;

- In the elastic phase, the interaction between soil and LDBP is close to being linear elastic. The applied load was predominantly transferred by friction in this stage (more than 90% of the total applied load). Plastic points start to form around the corner of the pile base by the end of the elastic phase. The ultimate pile load represents about 1.5–2.5 times the load at which the first plastic point was formed around the pile base. The factor of safety of 2–2.50 or greater will guarantee that the compressible soil is still in its elastic phase, and therefore these values (F.S: 2–2.5) are recommended to calculate the LDBP working load.
- A noticeable increase in the pile settlement rate was observed in the field measurements and numerical results during the mobilization phase. The percentage of the load transferred by the bearing was also increased in this phase according to the pile’s diameter and length. The soil skin friction was increased to achieve its peak value of the soil shear strength at pile settlement ranging from 1%D to 3%D. Plastic points were existed at the base and extended to a large part of the shaft.
- In the failure phase, the pile load transferred by friction tends to be constant or slightly decreased, and the applied load was predominantly transferred by bearing. Apparent failure was often observed through the large induced pile settlement at the end of this stage. Results of the parametric numerical models with different pile geometries and soil properties revealed that the plastic points were existed at the base and covered almost the whole length of the pile shaft.

In-situ measurements of the Alzey Bridge LDBP were utilized to evaluate the modified approach's accuracy. Excellent agreement was obtained between the field measurements and the calculated ones. Error percentages were 0.34%, 2.51%, and 5.02% for the ultimate friction, bearing, and total capacities, respectively. Furthermore, four in-situ LDBP loading tests and twenty numerical models were embraced to validate the proposed approach. The comparison between numerical results and the calculated LDBP ultimate capacities showed that the Modified Meyerhof method was able to estimate reliable values for the ultimate capacity of the LDBP. The error percentages ranged from 0.267 to 7.75%. However, more in-situ loading tests for large diameter bored piles loaded to failure should be utilized to assess the proposed method and evaluate its accuracy.

## Supplementary Information

Supplementary Information.

## References

[1] O'Neill MW, Reese LC (1999). Drilled Shaft: Construction Procedures and Design Methods.

[2] El-Nahhas, F. M., El-Mossallamy, Y. M. & Tawfik, M. M. Assessment of the skin friction of large diameter bored piles in sand. In *Proceedings of the 17th International Conference on Soil Mechanics and Geotechnical Engineering* (2009).

[3] Karabeliov K, Cuéllar P, Baeßler M, Rücker W (2015). System identification of inverse, multimodal and non-linear problems using evolutionary computing—Application to a pile structure supported on non-linear springs. Eng. Struct..

[4] Limkatanyu S, Kuntiyawichai K, Spacone E (2009). Response of reinforced concrete piles including soil–pile interaction effects. Eng. Struct..

[5] Küçükarslan S, Banerjee PK, Bildik N (2003). Inelastic analysis of pile soil structure interaction. Eng. Struct..

[6] Al-Atroush ME, Hefny AM, Sorour TM (2021). A parametric numerical study for diagnosing the failure of large diameter bored piles using supervised machine learning approach. Processes.

[7] Felleniius, B. H. Unified design of piled foundations with emphasis on settlement analysis. Honoring George G. Goble—“Current Practice and Future Trends in Deep Foundations.” In *Geo-institute Geo-TRANS Conference*, Vol. GSP 125, 253–275 (ASCE Geotechnical Special Publication, 2004).

[8] Weltman, A. J. *Pile Load Testing Procedures*. DOE and CIRIA. Piling Development Group and Construction Industry Research and Information Association. Report PG7. London (1980).

[9] Kulhawy, F. H. & Hirany, A. Interpretation of load tests on drilled shafts, Part 2: Axial uplift. In *Proceedings of the Foundation Engineering*, Vol. 2, 1150–1159 (ASCE, 1989).

[10] Eslami, A. & Fellenius, B. H. Pile capacity by direct CPT and CPTumethods applied to 102 case histories. *Can. Geotech. J.* **34**(6), 886–904 (1997). (12) Study on pile ultimate capacity criteria and CPT-based direct methods.

[11] Niazi FS, Mayne PW (2013). Cone penetration test based direct methods for evaluating static axial capacity of single piles. Geotech. Geol. Eng..

[12] Meyerhof GG (1982). Limit states design in geotechnical engineering. Struct. Saf..

[13] Charles WWN, Terence LYY, Jonathan HML, Wilson HT (2001). New failure load criterion for large diameter bored piles in weathered geomaterials. J. Geotech. Geoenviron. Eng. ASCE.

[14] Abu-Farsakh MY, Titi HH (2004). Assessment of direct cone penetration test methods for predicting the ultimate capacity of friction driven piles. J. Geotech. Geoenviron. Eng..

[15] Tawfik, M.M. *Assessment of Load Settlement Behavior of Large Diameter Bored Piles in Sand Under Axial Compression Loads*. Ph.D. Thesis. Faculty of Engineering, Ain shams university, 320–333 (2010).

[16] Zhihui W, Guoliang D, Weiming G (2020). Field and theoretical analysis of the response of axially loaded grouted drilled shaft in extra-thick fine sand. Can. Geotech. J..

[17] Zhihui W, Guoliang D, Weiming G (2019). Field study on post-grouting effects of cast-in-place bored piles in extra-thick fine sand layer. Acta Geotech..

[18] Zhihui W, Guoliang D, Weiming G (2018). Full-scale load testing of two large-diameter drilled shafts in coral-reef limestone formations. Bull. Eng. Geol. Environ..

[19] Liu NW, Zhang ZM, Zhang QQ (2013). Destructive field tests on mobilization of end resistance of cast-in-situ bored piles. J. Cent. South Univ..

[20] Zhang QQ, Li SC, Li LP (2014). Field study on the behavior of destructive and non-destructive piles under compression. Mar. Georesour. Geotechnol..

[21] Ching J, Chen J-R (2010). Predicting displacement of augered cast-in-place piles based on load test database. Struct. Saf..

[22] ECP 202/4 (2005). Egyptian Code for Soil Mechanics—Design and Construction of Foundations Deep Foundations.

[23] WHO (1990). German Association for Earthworks and Foundation Engineering.

[24] AASHTO LRFD (2005). Bridge Designs Specifications—SI Units.

[25] Meyerhof, G. G. Theory and practice of pile foundations. In *Proceedings of the International Conference on Deep Foundations* Vol. 2 177–186 (1986).

[26] Mullins G, Dapp S, Lai P, Dennis ND, Castelli R, O’Neill MW (2000). Pressure grouting drilled shaft tips in sand. New Technological and Design Developments in Deep Foundations.

[27] El Wakil AZ, Kassim M (2010). Bulging as a pile imperfection. Alex. Eng. J..

[28] Eid M, Hefny A, Sorour T, Zaghloul Y, Ezzat M (2018). Full-scale well instrumented large diameter bored pile load test in multi-layered soil: A case study of damietta port new grain silos project. Int. J. Curr. Eng. Technol..

[29] Eid M, Hefny A, Sorour T, Zaghloul Y, Ezzat M (2018). Numerical analysis of large diameter bored pile installed in multi-layered soil: A case study of damietta port new grain silos project. Int. J. Curr. Eng. Technol..

[30] Meyerhof GG (1951). The ultimate bearing capacity of foundations. Géotechnique.

[31] Meyerhof GG (1976). Bearing capacity and settlement of pile foundations. J. Geotech. Eng. Div..

[32] Al-Atroush ME, Hefny A, Zaghloul Y, Sorour T (2020). Behavior of a large diameter bored pile in drained and undrained conditions: comparative analysis. Geosciences.

[33] Kampitsis AE, Giannakos S, Gerolymos N, Sapountzakis EJ (2015). Soil–pile interaction considering structural yielding: Numerical modeling and experimental validation. Eng. Struct..

[34] El Gendy M, El-Arabi IA, Kamal MA (2014). Comparative analyses of large diameter bored piles using international codes. J. Deep Found. Inst..

[35] Phoon KK, Retief JV, Ching J, Dithinde M, Schweckendiek T, Wang Y, Zhang LM (2016). Some observations on ISO2394:2015 Annex D (reliability of geotechnical structures). Struct. Saf..

[36] Sommer H, Hambach P (1974). Großpfahlversuche im Ton für die Gründung der Talbrücke Alzey. Der Bauing..

[37] Ezzat M, Zaghloul Y, Sorour T, Hefny A, Eid M (2019). Numerical simulation of axially loaded to failure large diameter bored pile. Int. J. Geotech. Geol. Eng. World Acad. Sci. Eng. Technol..

[38] Wehnert, M. & Vermeer, P. A. Numerical analyses of load tests on bored piles, numerical models in geomechanics. In *NUMOG 9th: Ottawa, ON, Canada* (2004).

[39] Prandtl, L. Über die Härte plastischer Körper. In *Nachrichten von der Gesellschaft der Wissenschaften zu Göttingen, Mathematisch-Physikalische Klasse* 74–85 (Dieterichschen Buchhandlung, 2004).

[40] Reissner, H. Zum Erddruckproblem. In *Proceeding 1st International Congress for**Applied Mechanics* (eds Biezeno, C. B. & Burgers, J. M.) 295–311 (1924).

[41] Terzaghi K (1943). Theoretical Soil Mechanics.

[42] Vesic AB (1963). Bearing capacity of deep foundations in sand. Highw. Res. Rec..

[43] Touma, F. T., Reese. L. C. Behavior of bored piles insand. In *Proceedings of the ASCE* Vol. 100(GT7), 749–761 (1963).

[44] Franke, F. Pile foundations—Single piles. In *ECSMFE* Vol. 2, 83–102 (1976).

[45] Kamal ZA, Arab MG, Dif A (2016). Analysis of the arching phenomenon of bored piles in sand. Alex. Eng. J..

[46] Gilbert C (1951). Rankine and the theory of earth pressure. Géotechnique.

[47] Reese, L. C., O’Neill, M. W. & Touma, F. T. Bored piles installed by slurry displacement. In *Proceedings of the 8th International Conference on Soil Mechanics and Foundation Engineering* Vol. 24, 203–209 (1972).

[48] Rollins KM, Clayton RJ, Mikesell RC, Blaise BC (2005). Drilled shaft side friction in gravelly soils. J. Geotech. Geoenviron. Eng..

[49] Hernan G, Juan G (2001). Correlation between DLT and STL—Case histories. J. Geotech. Geoenviron. Eng. ASCE.

