# Supplementary material for: Modified Meyerhof approach for forecasting reliable ultimate capacity of the large diameter bored piles
Source: Sci Rep. 2022 May 20;12:8541. doi: 10.1038/s41598-022-12238-w (PMC9122945; doi:10.1038/s41598-022-12238-w)
Supplement: Supplementary file 1 — Supplementary Information. [file 41598_2022_12238_MOESM1_ESM.docx]

# APPENDIX A

**[a]** Case (4): Pile with Diameter of 0.60m **[b]** Case (5): Pile with Diameter of 0.70m

**[c]** Case (6): Pile with Diameter of 0.80m **[d]** Case (7): Pile with Diameter of 1.0m

**[e]** Case (8): Pile with Diameter of 1.20m **[f]** Case (9): Pile with Diameter of 1.50m

**[g]** Case (10): Pile with Diameter of 2.0m **[h]** Case (11): Pile with Length of 13.0m

**[i]** Case (12): Pile with Length of 19.0m **[j]** Case (13): Pile with Length of 26.0m

**[k]** Case (14): Soil with Young's modulus of 20000kN/m^2^ **[l]** Case (15): Soil with Young's modulus of 30000kN/m^2^

**[m]** Case (16): Soil with Young's modulus of 60000kN/m^2^ **[n]** Case (17): Soil with Young's modulus of 80000kN/m^2^

**[o]** Case (23): Soil with a friction angle of 10^0^ **[p]** Case (20): Soil with a friction angle of 15^0^

**[q]** Case (21): Soil with a friction angle of 30^0^ **[r]** Case (24): Soil with effective cohesion of 30kN/m^2^

**[s]** Case (25): Soil with Effective cohesion of 50kN/m^2^  **[t]** Case (26): Soil with Effective cohesion of 100kN/m^2^

**Fig. A** Comparison between the Numerical model’s results and the calculated settlement and ultimate capacity using the combined approach.

**Table A. Mechanical properties of four LDBP case histories**

|  | **Input** | | | | | | | **Results obtained from the Field loading tests** | | | | **Results calculated using the Modified Meyerhof Method** | | | | | | **Reference** | |
| --- | --- | --- | --- | --- | --- | --- | --- | --- | --- | --- | --- | --- | --- | --- | --- | --- | --- | --- | --- |
| **Case No** | **D** | **L** | **Soil Layer** | **C** | **Ø** | **K0** | **γ** | **P_ult_ FE** | **P_f_ FE** | **P_b_ FE** |  | **P_ult_ Equ** | | **P_f_ Equ** | | **P_b_ Equ** | |  |  |
| **Units** | **(m)** | **(m)** | **-** | **(kN/m^2^)** | **0** | **-** | **(kN/m^2^)** | **(kN)** | **(kN)** | **(kN)** |  | **(kN)** | | **(kN)** | | **(kN)** | |  | |
| 1 | **0.7** | **39** | 1-1 clayey silt | 14.8 | 28.7 | 0.520 | 18 | **7020** | **3674.68** | **3345.32** | 7233.56 | | 3872.438 | | 3361.118 | | Zhang et al., 2014 [49] | |  |
|  |  |  | 1-1 clay silt | 14.8 | 28.7 | 0.520 | 18 |  |  |  |  |  |  |  |  |  |  |  |  |
|  |  |  | 2-1sand silt | 13.5 | 29.2 | 0.724 | 19 |  |  |  |  |  |  |  |  |  |  |  |  |
|  |  |  | 3-1 silt sand | 9.6 | 30.4 | 0.699 | 19 |  |  |  |  |  |  |  |  |  |  |  |  |
|  |  |  | 3-3 silt clay | 17.2 | 9.2 | 0.840 | 18 |  |  |  |  |  |  |  |  |  |  |  |  |
|  |  |  | 4-1 silty clay | 60.3 | 15.2 | 0.738 | 18 |  |  |  |  |  |  |  |  |  |  |  |  |
|  |  |  | 5-1 silt clay | 31.4 | 22.5 | 0.617 | 18 |  |  |  |  |  |  |  |  |  |  |  |  |
|  |  |  | 6-1 gravel | - | 40 | 0.357 | 20 |  |  |  |  |  |  |  |  |  |  |  |  |
|  |  |  | 6-2 gravel | - | 40 | 0.357 | 20 |  |  |  |  |  |  |  |  |  |  |  |  |
| 2 | **0.8** | **39.8** | 1-2 Silty sand | 12.6 | 30.1 | 0.86 | 19 | **10000** | **6079.01** | **3920.99** | 10034.48 | | 6167.152 | | 3867.325 | | Zhang et al., 2014 [49] | |  |
|  |  |  | 1-2 Silty sand | 12.6 | 30.1 | 0.863 | 19 |  |  |  |  |  |  |  |  |  |  |  |  |
|  |  |  | 2-1sand silt | 13.5 | 29.2 | 0.72 | 19 |  |  |  |  |  |  |  |  |  |  |  |  |
|  |  |  | 3-1 silt sand | 9.6 | 30.4 | 0.69 | 19 |  |  |  |  |  |  |  |  |  |  |  |  |
|  |  |  | 3-3 silt clay | 17.2 | 9.2 | 0.84 | 18 |  |  |  |  |  |  |  |  |  |  |  |  |
|  |  |  | 4-2 Silty Clay | 54.9 | 15.8 | 0.72 | 18 |  |  |  |  |  |  |  |  |  |  |  |  |
|  |  |  | 5-2 Fine Sand | 15.3 | 27.7 | 0.53 | 18 |  |  |  |  |  |  |  |  |  |  |  |  |
|  |  |  | 6-1 gravel |  | **40** | 0.35 | 20 |  |  |  |  |  |  |  |  |  |  |  |  |
|  |  |  | 6-2 gravel |  | **40** | 0.35 | 20 |  |  |  |  |  |  |  |  |  |  |  |  |
| 3 | **1** | **47.6** | 1-1 Clayey Silt | 14.8 | **28.7** | 0.73 | 18 | **13320** | **8700.66** | **4619.34** | 13235.11 | | 8284.064 | | 4951.048 | | Zhang et al., 2014 [49] | |  |
|  |  |  | 1-1 Clayey Silt | **14.8** | 28.7 | 0.73 | 19 |  |  |  |  |  |  |  |  |  |  |  |  |
|  |  |  | 2-1sand silt | **13.5** | 29.2 | 0.72 | 19 |  |  |  |  |  |  |  |  |  |  |  |  |
|  |  |  | 3-1 silt sand | **9.6** | 30.4 | 0.49 | 19 |  |  |  |  |  |  |  |  |  |  |  |  |
|  |  |  | 3-3 silt clay | **17.2** | 9.2 | 0.84 | 18 |  |  |  |  |  |  |  |  |  |  |  |  |
|  |  |  | 4-1 Silty Clay | **60.3** | 15.2 | 0.73 | 18 |  |  |  |  |  |  |  |  |  |  |  |  |
|  |  |  | 5-2 Fine Sand | 15.3 | 27.7 | **0.53** | 19 |  |  |  |  |  |  |  |  |  |  |  |  |
|  |  |  | 6-2 gravel |  | 40 | **0.35** | 20 |  |  |  |  |  |  |  |  |  |  |  |  |
|  |  |  | 6-3 gravel |  | 34 | 0.44 | 20 |  |  |  |  |  |  |  |  |  |  |  |  |
| 4 | 1.2 | 16.5 | Medium to very dense Clayey Sand | 20 | 36 | 0.58 | 19 | 7200 | 4180 | 3020 | 7259.26 | | 4200.81 | | 3058.45 | | Hernan and Juan 2001 [50] | |  |
